# The role of patient and public involvement leads in facilitating feedback: “invisible work”

**DOI:** 10.1186/s40900-020-00209-2

**Published:** 2020-07-10

**Authors:** Elspeth Mathie, Nigel Smeeton, Diane Munday, Graham Rhodes, Helena Wythe, Julia Jones

**Affiliations:** 1 CRIPACC, University of Hertfordshire, Hatfield, AL10 9AB UK; 2 Patient and Public Involvement Panel, Cambridge University Hospital (CUH), Addenbrookes Hospital, Cambridge, CB2 0QQ UK

**Keywords:** Patient and public involvement, Feedback, Public involvement lead, Mixed methods

## Abstract

**Background:**

Health research in the UK requires patients, those with lived experience and members of the public to be involved in designing and shaping research: many of them have reported that their comments and suggestions are not always acknowledged, and they do not know if their input has been used or is useful. The benefits of feedback from researchers not only create motivation for further involvement but aids learning and development, as well as recording impact. The aims of this study were to improve the feedback experience of Patient and Public Involvement (PPI) contributors. Co-produced feedback processes were designed and implemented in order to change feedback from researchers to PPI contributors in six PPI groups in England.

**Methods:**

An explanatory mixed methods sequential study design was utilised with a comparative questionnaire survey (administered 20 months apart), interviews and a focus group with PPI leads, researchers and PPI contributors. Patient and Public Involvement contributors were involved from initial idea, study design, data analysis through to dissemination.

**Results:**

Co-designed feedback processes were introduced in five of the six PPI groups and there was an overall increase in the frequency of feedback over the period studied. The enablers and barriers to implementing feedback processes were identified, which included the importance of wider institutional level support. PPI leads need to have dedicated time and acknowledge feedback as part of their role. The importance of individual feedback processes designed by, and for each PPI group, rather than a generic one, was also identified as key to successful implementation.

**Conclusion:**

The role of the PPI lead is an important facilitator in improving feedback but can easily be overlooked and has been described as invisible. PPI leads can perform an essential bridging role between researchers and members of the public. This study has shown that PPI feedback processes can be implemented if they are part of embedded PPI with explicit expectations, facilitated by a dedicated PPI lead role with sufficient support and resources. The findings have implications beyond this particular study, particularly for those involved in undertaking and funding health and social care research.

## Plain English Summary

Members of the public play an important role by contributing to many aspects of health research. However, they seldom hear if their involvement has been useful, used or changed the research. This study aimed to fill the gap by improving feedback from researchers to those who have assisted them. Feedback can have a number of benefits for all involved: it can be motivating, aid learning and enable tracking of the impact the involvement has had. The term Patient and Public Involvement (PPI) contributor is often used to describe those involved in this way in health research.

This study included people from six Patient and Public Involvement groups, those who ran the groups and researchers. We carried out surveys (20 months apart), interviews and focus groups to gather views on feedback. The Patient and Public Involvement groups co-designed ways to improve feedback.

The findings revealed that feedback can be improved. However, it needs a co-ordinator/lead who has time and capacity and importantly support from the organisation in which the group sits. It was also found that group specific forms or processes were preferable to general ones. Those who run Patient and Public Involvement groups are key to bridging the relationship between researcher and Patient and Public Involvement contributor.

Significantly, this research also indicates that the role and work of those who coordinate Patient and Public Involvement groups has been undervalued: it was described as ‘invisible’. The importance of having enough resources, time and support is key to effective PPI and has implications beyond this particular study.

## Background

Although some Patient and Public Involvement (PPI) contributors operate independently and work directly with researchers, many are attached, belong to or are members of a PPI group usually facilitated by a PPI lead or co-ordinator. PPI groups are run in a variety of ways and operate in different settings; for example, within hospitals, universities, charities, carer and service user groups. A number of PPI groups have reported on their ways of operating, for example in palliative care and mental health [1, 2] and public member groups [3]. Fredriksson and Tritter [4] make the difference between PPI groups who take a generic ‘public view’ and others who focus on specific health conditions and bring their own experience to the research.

Researchers often make their first contact with PPI contributors via a PPI lead. This three-way relationship frequently starts when researchers begin to work with members from a group for the first time. There are no accurate figures on how many PPI leads exist within the United Kingdom in organisations such as Universities, hospitals and National Institute of Health Research (NIHR) roles but in 2016, 100 PPI leads attended an NIHR meeting in London [5]. At this meeting it was acknowledged that the PPI lead role is very varied and many learning needs were identified. The PPI facilitator role has been described as “a boundary spanner” [6] bridging between two worlds of academia and public [7] (p.124). Guidance and articles on PPI often assume that the relationship occurs directly between researcher and PPI contributor, whilst ignoring the role of many PPI leads who, at least in the beginning, initiate or facilitate the relationship.

Researchers working together with PPI contributors seek ideas, suggestions or comments whilst developing research. PPI contributors provide their comments to researchers (sometimes indirectly via the PPI lead) and then there is the opportunity for researchers to feedback on the contributions. These ‘feedback’ comments enable PPI contributors to know their comments have been received, whether they were used and if they were useful. Recent research found roughly one in five (19%) of PPI contributors said they had never received feedback [8] and a NIHR national survey in 2019 found a similar figure of 22% [9]. Feedback is important to PPI contributors for motivation, for building confidence and for learning and development [8]. It is also respectful and an acknowledgement that peoples’ contributions are valued and are not a waste of their time. In addition, feedback enables researchers to reflect and record the impact of PPI within the study.

Patient and Public Involvement (PPI) is now recognised as an important component of health and social care research [10, 11] and international as well as national guidelines, frameworks and standards are available [12–15]. A growing number of research funders and journals now require PPI to be included and reported [16, 17]. With reporting of PPI processes and mechanisms, there is more understanding of what works for whom, in what circumstances and why [7, 18–20] . The UK National Standards for Public Involvement were launched in 2018 and updated in November 2019 to “improve the quality and consistency of public involvement in research” [13]. One important and often neglected area is improving the experience of those involved in PPI and, within this, the role of the PPI lead.

Attempts have been made to identify “if, when, where and how involvement brings benefits” and define the impact of PPI [20] (p.2). PPI impact can be in terms of changes to the research, but can also have an impact on the researchers, PPI contributors and the wider community [21]. Although research cited in the literature has called for increased evaluation of PPI impact, [19] there has been a recent focus on ‘improving’ the process and experience of PPI rather than focussing solely on ‘proving’ [22]. The focus on improving feedback is one way of identifying some of the impact of PPI. The rationale for this research came from PPI contributors who were dissatisfied with the quantity and quality of feedback from researchers and who wanted to improve the situation.

The first stage (2016–2017) of the research study explored the definition, frequency and importance of feedback and is reported in Mathie et al. (2018) [8, 23] and the second stage (2017–2018) (reported in this paper) designed and implemented local feedback processes or tools within six PPI groups. The aim of this paper is to describe the introduction of co-designed PPI feedback tools and processes, with a particular focus on the role and remit of the PPI lead.

## Methods

### Study design

**Fig. 1:**
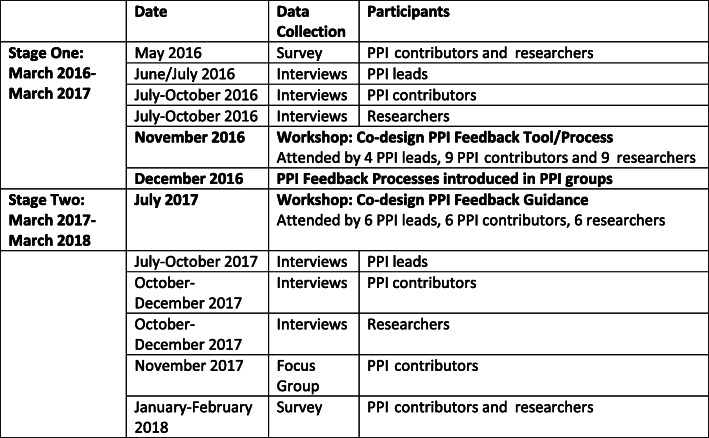
**Timeline of Data Collection: Stage One and Two**

An explanatory mixed method sequential study design was utilized with a baseline and follow-up questionnaire survey, semi-structured interviews (at two time points) and one focus group. The methodology builds upon the on-line survey and interviews reported in Mathie et al. [8]. The timeline of the methodology is given in Fig. 1. A survey of PPI contributors and researchers was undertaken at the beginning of the study (Stage one) and again at the end (Stage two) (see Additional file 1). The aim of the surveys was to explore the changes, variation, types, importance of, and satisfaction with feedback within six PPI groups: it was completed by both researchers and PPI contributors. The baseline survey took place in May 2016 (Stage one) and a follow-up survey took place in January/February 2018 (Stage two).

A co-produced feedback tool/process was developed in November 2016 and introduced within the PPI groups in December 2016. The follow up survey took place when feedback processes had been in place for approximately 12 months.

In addition to the surveys, in-depth information about feedback was collected from the PPI leads, PPI contributors and researchers through interviews and also one focus group with PPI contributors (see Additional file 2). These were organised in Stage one before the PPI feedback processes were introduced and then repeated approximately 12 months later. Interviewees were initially identified from the Stage one survey.

PPI contributors played a major role in this study from initiating the research idea, data collection, analysis to dissemination: this involvement has already been reported, [8, 23]. PPI contributors were involved in this second stage through input in meetings, co-designing the PPI feedback tools and in the co-produced ‘Guidance for Researchers: PPI Feedback’ [24]. The aim of PPI in this study was to ensure the research addressed concerns identified by the PPI contributors and they were involved in co-designed materials which would be used to implement feedback (see Additional file 3 GRIPP2 checklist).

### Setting and participants

Participants were purposively selected to include six PPI groups in one region of England. All 227 PPI contributors and 316 researchers who had used the PPI groups in the last 18 months were sent an invitation to take part. The surveys were distributed by the PPI leads and were completed anonymously. All eleven PPI leads who facilitated the six PPI groups within the time period were invited to take part in interviews. The six groups consisted of a mixture of public and patient panels, based within Universities or National Health Service organisations.

### Data collection

This was a mixed methods study, utilising different methods of data collection with a quantitative survey followed by qualitative interviews and a focus group to capture a broad range of views and then follow up in more depth. This sequential approach also facilitated the recruitment of a sub-sample of survey respondents (researchers and PPI contributors) who were interviewed at Stage one [8]. The interview participants were purposively selected using a maximum variation sampling approach (PPI group, demographic characteristics such as age and gender, PPI and feedback experience). All the researchers and PPI contributors who were interviewed in Stage one were invited to a second (repeat) interview approximately a year later in order to determine whether changes had been made following the introduction of feedback processes. In order to capture the experience of those who had used the feedback processes, more researchers and PPI contributors were recruited to Stage two (via the PPI lead). All PPI contributors were offered the opportunity to take part in a focus group to understand their views about changes in feedback or, if the location and date was not convenient, a one to one interview using the same topic guide (see Additional file 2). The rationale for offering different data collection methods to PPI contributors (focus group or interview) was to be flexible and inclusive. All PPI leads from the six PPI groups were interviewed (face to face or by telephone) at the first stage and re-interviewed (if still employed) towards the end of the study, with several new PPI leads; these interviews have not been reported previously [8]. The interview schedule focussed on the implementation of feedback processes.

### **The sample**

As shown in Table 1, in Stage one 101 participants completed the survey questionnaire and 81 in Stage two. Forty-nine interviews were conducted in total, with 11 PPI leads, 16 PPI contributors and 13 researchers. Seven PPI contributors participated in a focus group.

**Table 1:** **Numbers of Participants**

	Stage one (2016–17)	Stage two (2017–18)	Total Interviews
***Survey***			
PPI contributor	68^a^	46	
Researcher	39^a^	35	
***Total Survey Participants***	***101***^a^	***81***	
***Interviews***			
PPI Leads	8	6^b^	14
PPI contributors	9^a^	12^b^	21
Researchers	6^a^	8^b^	14
***Total Interviews***	**23**	**26**	**49**
***Focus Group***			
PPI Contributors	0	7 (1 focus group)	

^a^Data reported in Mathie et al. (2018) [8]

^b^Repeat interviews, 3 PPI leads, 5 PPI contributors, 1 researcher

Interview Quote IDs: .01 = Stage one interview .02 = Stage two interview

### Data analysis

The quantitative survey data was transferred (via Excel) to SPSS (version 23.0) and paper copies were manually entered. Descriptive statistics were obtained to summarize the attributes of the participants and provide an overview of the variables. The qualitative open questions from the survey, the interview data (49 transcripts) and one focus group transcript was entered into NVIVO (Version 11). Analysis was carried out both within and between themes and care was taken to avoid decontextualization by referring back to the original data, to make sure the context of the theme or quote was maintained. Attention was given to analyse views from the different groups of participants (PPI contributors, researchers and PPI leads and PPI groups as a whole), those who completed both first and repeat interviews and those who only completed one interview, noting and comparing differences in views, themes and data collection methods. The integration of quantitative and qualitative data occurred at the interpretation level of analysis [25].

### Ethics

The study received approval from the Proportionate Review Subcommittee of the North West – Liverpool Central Research Ethics Committee (REC 16/NW/0245; IRAS 203158) in April 2016 and an amendment for a further year was approved in March 2017.

## Findings

This findings section draws particularly on the data from Stage two and the themes which focus on feedback processes; the setting up of feedback processes, co-designing feedback processes, any noted changes in feedback, the enablers and challenges to feedback and lastly, the role of the PPI lead in the feedback process. Findings on PPI contributors’ and researchers’ attitudes to feedback have been reported previously [8]. Findings are presented from the integration of data from all three sources of data collection methods.

Although the samples at Stage one and two share similar characteristics in terms of age group, gender and employment, the degree of participant overlap between the two survey samples is unknown. Ten (22%) PPI contributors in the Stage two survey told us they had completed the previous survey and 3 (9%) of researchers; however, it was not possible to match these participants due to the design of the surveys.

### Introducing feedback processes

At the start of the study, all six PPI leads stated that they did not have routine feedback processes in place for their PPI groups and that feedback to PPI contributors could be improved. As part of the research study, a workshop was held which gave the opportunity for each PPI lead, up to two researchers and two PPI contributors from each of the PPI groups to work together to decide how they were going to improve feedback (see [23]). A total of 4 PPI leads, 9 PPI contributors and 9 researchers attended the workshop which was facilitated by research team members (Fig. 1). PPI feedback processes were co-designed by the PPI groups. The PPI contributors involved in this study also felt it important to co-design some generic Feedback Guidance for researchers [24] and another face to face workshop was organised to start developing this guidance and attended by 6 PPI leads, 6 PPI contributors, 6 researchers and a Senior Advisor from INVOLVE. This completed Guidance is an important research output of the study, it has been used subsequently by the individual PPI groups but also promoted and used widely nationally and internationally [26, 27].

This rest of this section covers particular themes relating to feedback processes: the PPI groups and feedback processes at the start of the study; the co-design of feedback processes; any changes in feedback; the suggested enablers and challenges to improve feedback and the role of the PPI lead is discussed and considered.

### Description of the PPI groups and feedback processes

The PPI groups varied in size and focus; four were based within National Health Service (NHS) organisations and two within Universities. The six groups all worked in slightly different ways with researchers, and they varied in terms of PPI lead allocated time, frequency of contact and size of group (see Table 2). As part of the consultation process, all of the six PPI groups circulated research documents to their PPI contributors for comment (by email or in paper format) and invited comments via the same method of distribution, as well as face to face meetings with the majority of correspondence via the PPI lead. Some PPI groups held discussion groups or panel meetings to bring researchers and PPI contributors together. Face to face group PPI group meetings ranged from monthly to every 6 months and some were called “as and when needed”.

**Table 2:** **Description of Patient and Public Involvement Groups**

**PPI Group**	**A**	**B**	**C**	**D**	**E**	**F**
**Approximate number of PPI contributors**	25	70	30	20	70	15
**PPI Leads (working days)**	4 days a week	5 days a week	Variable	1 day a week	4 days a week (term time only)	4 days a week
**Length of time in current PPI Lead role**	Maternity Cover < 1 year	1 year (leaving)	Temp cover (4 years)	1 year	10 years +	6 months
**Type of panel**	Condition specific: mental health	Open, patient panel	Open, public panel	Restricted, public panel,	Open, public panel	Open, public panel, 2 year fixed term

In three of the six groups the PPI leads reported that they already had information for researchers which included advice about PPI feedback. However, this information was not routinely given to researchers: although the majority of PPI leads said the expectation of feedback was verbally outlined to researchers. One of the PPI groups already had a PPI Feedback form for researchers to complete but it was not routinely used. Another PPI lead said she had previously tried to use feedback forms but they were not completed and a further PPI lead described the feedback process as ‘ad hoc’.

### Co-designing feedback processes

As part of the study, five of the six PPI groups co-designed and tried to implement some form of feedback process. A joint workshop was held which included members from the six PPI groups to start the co-designing process. A number of ideas were discussed together, including: i) a simple, short ‘prompt’ to remind the researcher to provide feedback, ii) a structured form, which the researcher completes detailing if any changes were made and if not, why not and iii) an examination of the current research process and identification of areas for feedback. Three of the five groups chose to work on a structured Feedback form (one example is given in Additional file 4), whilst one group drew out the research process (including stages for potential feedback) on flip chart paper and another group suggested a simple spreadsheet. Examples from two of the PPI groups who used structured Feedback forms are given below.

*Example 1:* The PPI lead receives requests from researchers to make comments on their research. The PPI lead sends out an electronic email to all PPI contributors to ask for their comments on the research with a deadline/return date. All the comments from PPI contributors (which can be up to 12 individual comments) are then collated by the PPI lead into one document and these are then sent to the researcher with a feedback form. The feedback form is electronic. Once the researcher has considered the comments and decided on any subsequent amendments or changes, the researcher fills in the feedback form and sends it back to the PPI lead. The PPI lead then collates all researcher comments (from different projects reviewed by the PPI group each month) into one monthly spreadsheet which is shared with the PPI group via an on-line newsletter. One PPI contributor described how they received feedback;

*“she sends out the [monthly] newsletter and it’s a model for all other research and PPI groups to follow really because it’s so good, it’s done as a link to a Google document or something or other, opens up and says, this is the name of the study, this is the name of the researcher and this is their comment [feedback]” (PPI Contributor 29.02).*

*Example 2:* Researchers come to the PPI group and present to PPI contributors at a quarterly face to face group meeting which is run by the PPI lead. Research documents are sent round beforehand. During the meeting the PPI contributors make comments or send them in separately afterwards (for those who cannot attend). The researcher is then asked by the PPI lead to complete a feedback form on what actions they took following the comments from the PPI group. The researchers’ comments are collated and given back to the PPI members verbally, on paper and electronically via a spreadsheet at the next meeting. Researchers can also seek comments in between meetings; again, the feedback is provided at the quarterly meeting.

### Changes in feedback processes

The remainder of the findings section describes any changes in feedback following the implementation of feedback processes. As reported earlier, at the start of the study all of the groups admitted they did not have any routine feedback processes. The follow up (repeat) interviews with PPI leads revealed that the new feedback forms had helped to formalise the process;

“*I think that at first it probably was happening but it wasn’t being documented, so I think that's the main thing. So I have now used the tool.... it was good to kind of use it, and I think it’s not necessarily been documented in that way before. So I think it was happening, but we maybe weren’ keeping track of it quite so well, and I think we let it often happen slightly more ad hoc and informally in person, it’s quite good to have something written down*” (PPI Lead 04.02)

The PPI leads agreed the new feedback processes helped to organise the impact information into one place which was then easily accessible.

*“I think the feedback form is a way of helping us record this information because I have all this information either in writing or verbally but its lost in the email account or its lost in the documents we have for each project but it’s not recorded and it’s not used in a way that we can show what has happened as a result of the work we have been doing and we can use all this information as evidence*” (PPI Lead 08.02)

“*I think the feedback tool will really now be an important part of the process of recording impact from now, that was lacking before*” (PPI Lead 09.02)

The follow-on survey showed that overall 43% of PPI contributors reported that there had been an improvement in PPI feedback in the last year (51% said no change), and 38% of researchers believed that their PPI feedback had improved (55% said no change). The baseline and follow-up survey also showed that since the feedback processes had been introduced there had been an increase in PPI contributors reporting they always received feedback (16% to 38%) and a decrease in those who said they ‘never’ received feedback (19% to 7%) (Table 3).

**Table 3:** **Frequency of Feedback Received by PPI Contributors**

	**May 2016**	**January/February 2018**
	n (%)	n (%)
Always	10 (16)	17 (38)
Sometimes	40 (65)	25 (56)
Never	12 (19)	3 (7)
**Total**	**62 (100)**	**45 (100)**

Chi-squared test for trend *P* = 0.006

In addition, there had been an increase in researchers reporting that they always gave feedback (45% to 65%) and no participants reported ‘never’ giving feedback in the second survey (Table 4).

**Table 4:** **Frequency of Feedback Given by Researchers**

	**May 2016**	**January/February 2018**
	n (%)	n (%)
Always	17 (45)	22 (65)
Sometimes	17 (45)	12 (35)
Never	4 (11)	0 (0)
**Total**	**38 (100)**	**34 (100)**

Chi-squared test for trend *P* = 0.049

In terms of frequency of feedback from researchers there was some variation across the six groups. The change in percentage of contributors reporting always receiving feedback varied from an increase of 46% for one group to a decrease of 25% in another (where feedback processes were not successfully implemented and the PPI group became less active during the study period). These differences are difficult to interpret due to the small numbers involved in each group. A similar variation in change was seen in the percentage of researchers reporting always giving feedback, although for one group there was an increase of 88%. There was a much smaller improvement in reported satisfaction ratings, but the findings were not significant.

The second round of interviews (Stage two) with PPI contributors highlighted some positive experiences of improved feedback over the previous 12 months;

“*Well I think it’s definitely improved, yes, hmm, yes because we used to get very little … ..but now I think we’re getting it, it’s now got in, embedding more into the department and into the research. So I think they are more cognisant of trying to give something back*” (PPI Contributor 04.02)

*“But it’s working very well with [PPI lead], whatever she’s doing with the researchers is working very well because they seem, when you read the comments that the researchers have fed back, you get the impression that they’re being genuine and not just going through another tick-box exercise. And, you know, when it comes to being part of the reflective process of research, I suspect that researchers get a certain amount of doing the feedback and it’s not necessarily a drain but a way of them reflecting on what has happened and saying, “Yes, there’s value to that,”” (PPI Contributor 29.02)*

There were also those who were part of the same group who noticed moderate change.

*“The impression I got, it wasn’t”, I don’t remember any sort of excitedness of “Wow, I’m getting lots of feedback now, it’s fantastic since the tools been introduced”, sort of moderate kind of “yeah, we are getting some feedback*” (PPI Contributor 07.02)

These quotations demonstrate that even though overall the feedback process had been improved, perception and experience of feedback is very individual.

### Enablers and challenges for implementing feedback processes

The findings identified that the feedback process needs to be relatively easy for both PPI leads and researchers. In the interviews, researchers reported finding the feedback forms fairly straightforward to complete (although formatting issues were reported) and PPI leads felt it was important that feedback became part of the PPI process;

“*it needs to be integrated within the general guidance and protocol of how the group works and the expectations we have of researchers and making that much more transparent right from the beginning*” (PPI Lead 09.02)

and feedback needs to become part of researchers’ expectations;

“*I think there has been a change both in training and attitude, and the role and attitude of the researchers”* (PPI Contributor 04.02)

The importance of the PPI group owning their individual PPI processes and feedback forms (rather than developing generic versions as originally suggested by some of the PPI contributors) became very clear as the following quotation demonstrates;

“*I think that fact that they have been involved in developing it from Day 1 means that they have ownership of it. They [PPI contributors] have made quite a few changes when we were developing it… .we agreed we would run with it for a few months and then re-visit and see if we needed to make any changes with it… .everyone had an opportunity to feed into its development… .when that happens you always have more ownership from the whole group with a new tool or form or way of working*” (PPI Lead 09.02)

Another essential factor for keeping the feedback relevant to those involved was the refining, reviewing and iterative process. The timing of when to administer feedback forms was also raised as PPI impact may not be immediate and one researcher felt that feedback needed to be ongoing;

“I *think it’s really good to have one [feedback form] immediately because you get your kind of gut reaction, your immediate feedback. But I think something like three months later, or six months later, where you’ve actually got a better understanding of why some of those comments might be useful and how that might shape your research*” (Researcher 01.02)

Overall, researchers who took part in this study were positive about the feedback process. However, it was also acknowledged that feedback takes time, there are budget constraints, researchers are subject to short contracts and some researchers do not know feedback is expected [8].

### The role of the PPI Lead

The six PPI groups who took part in the study all had the support of a PPI lead or co-ordinator and their time allocated for PPI ranged from 1 day to 5 days a week with some having additional administration support (Table 2). The PPI leads had varying roles which were either as academic researchers who held the additional PPI lead role alongside their regular work or those who were employed solely for this purpose. The backgrounds of the PPI leads were varied; voluntary sector, law, social services and medical education quality control. Two of the groups had been running for over 10 years and one of the PPI leads had been also been in post for a similar length of time. The remaining PPI leads were much newer to post (1–2 years). In total, 11 PPI leads were in post from the six PPI groups over the two-year study period. During the study, one PPI lead went on maternity leave and two left their posts, of the 11 all were female except one. The PPI leads role includes connecting the researchers to the PPI contributors enabling both parties to have a greater understanding of each other’s role, creating an opportunity for PPI contributors to become members of trial and project steering groups and assisting researchers to facilitate group meetings.

The role of the PPI lead in facilitating communication between researchers and PPI contributors was viewed as being central in supporting the feedback process. Many individual PPI contributors recognised the importance of the PPI lead role in improving feedback;

*“We are certainly getting quick feedback from researchers in projects presented to the [group] meetings in last year, through the PPI coordinator and more response in general over the last year. The importance of the role of the coordinator is reflected in these improvements and demonstrates how key is the role” (PPI Contributor 101: Survey 02)*

*“it’s improved enormously since [xx] is in post. I think she’s [PPI lead] made huge efforts to relate to PPI representatives … give them good feedback” (PPI Contributor 06.02)*

*“she’s an excellent person updating us and everything. But again, feedback isn’t perfect but it isn’t bad either” (PPI Contributor 03.02)*

In terms of a challenge at the organisational level, respondents from two of the PPI groups mentioned lack of resources and capacity. One PPI lead said "*I haven’t really got any allocated time for it … ..I haven’t got a protected time on a weekly basis*" (PPI Lead 09.01) which was in contrast to another PPI lead who was full time and felt she had "*great freedom*" (PPI Lead 10.02). One PPI contributor praised the commitment of the PPI lead, but referred to the wider organisational structures that prevented feedback from happening. The PPI contributor described the reduction in resources in terms of staffing time and the lack of infrastructure;

*"if the organisation and institution don’t take it [feedback] on board and don’t support the individual who’s doing it, it isn’t going to happen … … *

* … ..you can’t take that time out to support those individuals, to give them that feedback … .unless your employer, your institute … .your job description, allows you to do that*" (PPI Contributor 05.02)

In situations where PPI contributors are members of a PPI group, having a dedicated PPI lead appeared crucial for successful feedback. The PPI lead needed to be flexible enough or have sufficient authority to see feedback as part of their role. The PPI leads reflected on their roles;

“..*it’s* [feedback] *an important part of the job, and I think in terms of my, you know, the role description for this job and things, it very much fits in with … .making those particular, changing that, the relationships between the researchers and the [PPI group], I think, but, yeah, I don’t think it’s out of place, even if it is a bit of work sometimes*” (PPI Lead 04:02)

“*I was taken on to coordinate and develop the Panel, so that has been the focus of my role, so it’s got, it’s got two strands, it’s got where I am recruiting and maintaining, establishing relationships with current members, and also promoting the work of the [PPI group] to the public, not necessarily to recruit them into the [PPI group], but to help them to realise that this goes on, because I think people will feel good to know that this happens even if they can’t give their time to do it at the moment … …*

*...and then the other side is a customer service to the researchers, and I had an interesting conversation about customer service, because I don’t think that the [PPI group] are a service, but I think my particular role is the service role, and so I think it, I think of it as being like the invisible, or as invisible as possible, bringing the two sides together. But there are some, some elements where I am more visible and establishing relationships is one*” (PPI Lead 10.02)

The ‘invisible’ metaphor is very revealing of the role that some PPI leads believe they have to play in managing relationships between researchers and PPI contributors. The same PPI lead explained how she introduced the idea of feedback to researchers, as something they had to do anyway;

“*my point is if you’re going to fill this in [PPI] for a funding application, you’re going to have to do that anyway so it would only be a question of letting us know what you’re going to do*” (PPI Lead 10.02)

Interestingly, another PPI lead used the word ‘service’ to describe the work of PPI contributors and a reason why researchers might be reluctant to provide feedback;

“*one researcher said people are providing a free service so it feels very strange to start scoring them, or marking them, on how well they did... so I think researchers are also uncomfortable providing feedback” (PPI Lead 02.01)*

One researcher used the term ‘service’ to describe the PPI group and this researcher described going through a PPI lead as quite “anonymous”. One potential danger of the role of the PPI lead is that it removes the researcher from the direct relationship with PPI contributors; for example;

“*the whole thing [going through a PPI group] just feels very anonymous, because you’ve got no sense of who the people are that are responding to you, you've got no way of, you don’t know, so it’s kind of, it’s almost like you put your thing in and out comes the answer, I think before I’d attended your [meeting] I hadn't really, which is terrible, but I hadn’t, I hadn’t thought about who was involved or what they were doing or what the process was on the other side, because you just get fed back, yeah, you just get fed back*” (Researcher 04.02)

This view was unique and not a view held by other researchers. However, it is acknowledged that the process of introducing a form does not change attitudes or necessarily improve relationships between PPI contributors and researchers. However, the “feedback process” when introduced by a PPI lead, along with raising expectations of the researcher, did enable regular feedback which was seen by one group as a catalyst to change and alongside other factors, could lead to culture change;

“*I think it [feedback process] act as a catalyst and maybe change things quite a lot*” (PPI Contributor 06.02)

“*one set of doctors even came back and said ‘this has not only changed my information sheet, it’s going to change how I speak to patients on the ward’”* (PPI Lead 10.02)

The process of collecting feedback also helped to identify and record impact, which can then be fed back to PPI contributors and enable them to see the benefits of their involvement.

## Discussion

This research confirmed that PPI leads are vital in order to improve the overall experience of PPI contributors in regard to feedback. Their role in implementing feedback processes is key to the overall experience. Leadership has been identified as a crucial part of PPI [28] but the role of the PPI lead has been given less attention to date. Although we have used the term PPI lead in this paper as a commonly recognised term, facilitator or co-ordinator might reflect the role more accurately. The job description, role of a PPI lead, varies and it has been recognised there are many diverse career paths with ‘no qualification’ for public involvement or no recognised benchmark [5, 29]. The role of the PPI lead is key to facilitating PPI and feedback, and it is often “invisible” work as described by one PPI lead. The ‘invisible’, largely unrecognised role of the PPI lead, of whom the majority in the study were female draws a parallel with the sociological literature of housework and caregiving [30, 31] and gendered roles where work is often unrewarded and undervalued. There is limited background data on the gender of PPI leads, although a 2014 report confirms that females were in the majority [29].

The importance of the PPI coordination work was recognised in the RAPPORT study with coordinators often bridging two worlds: research and lay [7]. The skills needed to facilitate between the needs of both researchers and PPI contributors are numerous [6, 32] and Wilson et al. (2015) found Patient and Public Involvement was more strongly embedded if there was a person with a designated responsibility for co-ordinating and facilitating PPI [7]. Our feedback research raises the issue of whether PPI feedback is about the exchange of knowledge, a learning process, a service or a combination. PPI leads can be important communication facilitators. However, care must be taken not to create a distance or disparity between the expectations and experience of the PPI contributor and the researcher. Although this study has focussed on PPI leads who facilitate PPI groups, feedback is equally important for researchers who are working directly with PPI contributors.

Feedback is not unique to research and parallels can be drawn with education. PPI contributors may want different sorts of feedback and some are content with an acknowledgement for their contribution [8]. However, for those who see feedback as promoting learning and development the concept of single-loop, double-loop, closing of feedback loops and longer term feedback spirals in education maybe useful [33]. It is not suggested that that PPI contributions are ‘work’ as such to be judged on quality but the learning is mutual and on-going communication between researcher and PPI contributor focusses on how to work together [34]. In trying to address the lack of feedback [35, 36] we understand more about the process of PPI, motivations and relationships between PPI contributors and researchers. The importance of emancipatory knowledge, valuing and strengthening personal gain and empowerment within PPI processes has been recognised as a key part of the moral or ethical rationale for PPI [37].

At a local level, the feedback processes which were developed and co-designed as part of this study were successful largely because the groups felt ownership and PPI leads had capacity, organisational support and individual autonomy to introduce them. Although the processes and forms were very similar, it appeared to be important that each group developed and ‘owned’ their own way of working. The success of implementation can be attributed to the co-design by PPI contributors, researchers and PPI leads working together and it was also helpful having members from different groups sharing ideas, which happened during the workshop. This suggests that these local groups preferred to find their own solutions and refine their own ways of working, whilst appreciating the opportunities to learn from others and being guided by more general National guidelines [13]. Initially, it had been suggested by the PPI contributors that we develop one ‘generic’ form: however, as we worked together it increasingly became apparent that in order for it to be successfully implemented each PPI group should develop, adapt and modify their own process. Greenhalgh and colleagues in their systematic review of PPI frameworks suggest adapting existing evidenced-based resources to local circumstances and consider “co-designed workshops to generate a locally relevant and locally owned framework” [15] which is what worked well in our study.

Our study did not collect information about how the PPI groups were funded or resourced but the importance of adequate capacity to implement feedback processes was identified. Researchers who seek involvement before specific project linked funding is available, often rely on existing, established PPI groups who may be centrally funded. Given the recent expectations around wider PPI to include engagement, community and co-production [38] sufficient resources and organisational structures for PPI are particularly significant.

There has been critical debate about PPI in the literature; “*lack of clarity on what PPI is (or might be)”* and “*at its worse …*. *PPI runs the risk of being insignificant, tokenistic, and overly managerialist*” [39]. However, in trying to understand the process of PPI, trying to make improvements and attempting to introduce routine processes, it is suggested this can be the start of embedding wider, good PPI practice. When feedback becomes part of the process of researchers and PPI contributors working together, feedback can make PPI more visible by illustrating the impact of PPI on the aims, as well as the methodology and conduct of the research. Although PPI is a mandatory part of health and social care research in the UK, it is important to remember that some researchers are still reluctant to engage with PPI, with one recent research paper reporting that only 60% of researchers say “I want to do it [PPI]” [40]. The end of this feedback research study came at the same time as the launch of the UK National Standards for Public Involvement (March 2018) and the ‘Guidance for Researchers: PPI Feedback’ developed as part of this study was named as a resource for the communication standard [13, 24]. Wider messages about feedback and communications between researchers and PPI contributors were publicised at this time and reinforced the feedback results [41]. A number of groups, charities, organisations (nationally and internationally) now use the Guidance for Researchers: PPI feedback in their practice, training or on their websites [26, 42–45] and these findings suggest PPI feedback becomes an embedded part of PPI practice.

## Limitations

It is acknowledged that these findings are based on a small number of participants and the findings must be interpreted with caution. Bias may be an issue as those who took part may be atypical of the wider population. Although the samples share similar characteristics, the degree of participant overlap between the two survey samples is unknown. Hence, changes in response between the two surveys cannot be assessed at the level of the individual. The study was originally funded for a year and so the initial period to evaluate the intervention was short; however, once there was further funding the evaluation continued for a second year. There were no specific ‘young people’ PPI groups involved in the study but it reasonable to assume the role of the PPI lead would also be very important.

## Conclusion

The paper has highlighted the importance of the PPI lead role in implementing feedback. This study has demonstrated that working in a collaborative manner with PPI leads, PPI contributors and researchers is important to the success of feedback and to the whole experience of being involved in the PPI process [23]. Some of the PPI groups involved in this study have successfully introduced new feedback processes which are still being used. Despite some of the PPI leads in this study leaving their jobs, the PPI co-designed feedback processes which were introduced have started to become embedded and been picked up by the successor PPI lead. The importance of locally co-designed processes is clearly important and has contributed to successful implementation. However, those groups who are not as supported in terms of a dedicated PPI lead, time and resources, have been less successful in introducing or maintaining feedback processes to improve the experience for PPI contributors. Feedback to those who are involved in research is a key area for improving communication, recording impact and is relatively simple to address. Implications of this study going forward are to highlight the role of the PPI lead in the feedback process and also to recognise the responsibility of researchers to work with PPI contributors from the beginning of research. However, PPI needs to be well supported and resourced by the organisations within which PPI groups sit, to enable PPI to become part of everyday practice. Resources and financial models of sustainability of PPI are ongoing concerns and the findings have implications beyond this particular study.

## Supplementary information

**Additional file 1.** Survey.

**Additional file 2.** Topic guide.

**Additional file 3.** GRIPP2 checklist.

**Additional file 4.** Researcher Feedback Form 2018.
