# Supplementary material for: The role of patient and public involvement leads in facilitating feedback: “invisible work”
Source: Res Involv Engagem. 2020 Jul 10;6:40. doi: 10.1186/s40900-020-00209-2 (PMC7353750; doi:10.1186/s40900-020-00209-2)
Supplement: Supplementary file 1 — Additional file 1. Survey. [file 40900_2020_209_MOESM1_ESM.pdf]

Copy of Completing the Feedback Cycle II: Survey of PPI Representatives

Questions 1-5 will help us describe our survey respondents

1. Have you previously completed an online survey about PPI feedback (May 2016)?

- ◯ Yes
- ◯ No
- ◯ Don't know

2. What gender do you consider yourself to be?

- ◯ Male
- ◯ Female
- ◯ Indeterminate

3. Which age group do you fit within?

- ◯ 16-25 years old
- ◯ 26-35 years old
- ◯ 36-45 years old
- ◯ 46-55 years old
- ◯ 56-65 years old
- ◯ 66-75 years old
- ◯ 76 and over

4. What is your marital status?

- ◯ Single
- ◯ Married
- ◯ Living together
- ◯ Widowed
- ◯ Divorced / separated
- ◯ In a relationships but none of the above
- ◯ I don't wish to say

5. What is your employment status? You may select more than one.

- ☐ Employed (PT/FT)
- ☐ Unemployed
- ☐ Student
- ☐ Retired
- ☐ Carer
- ☐ Other (please specify)

6. Which PPI group are you attached to? You may tick more than one.

- ☐ Group 1
- ☐ Group 2
- ☐ Group 3
- ☐ Group 4
- ☐ Group 5
- ☐ Group 6
- ☐ Group 7
- ☐ Other (please specify)

7. Approximately, how long have you been doing this role as Patient and Public involvement?

- ◯ Under 6 months
- ◯ More than 6 months - 1 year
- ◯ More than 1 year - 2 years
- ◯ More than 2 years - 3 years
- ◯ More than 3 years - 4 years
- ◯ More than 4 years - 5 years
- ◯ More than 5 years - 10 years
- ◯ Over 10 years
- ◯ Other (please specify)

8. What stages of the research process have you been involved in? You may tick more than one.

- ☐ Priority setting (coming up with a research question/helping decide what research to do)
- ☐ Design of research (input into design and commenting on protocols, patient information sheets)
- ☐ Management of research (Advisory Boards / Steering Committees)
- ☐ Undertaking research (carrying out interviews, data analysis)
- ☐ Dissemination of findings (talking/writing about the results)
- ☐ Co-researcher
- ☐ Co-applicant
- ☐ Other (please specify)

9. At what stage of the research cycle do you think PPI is most useful?

- ◯ Priority setting (coming up with a research question/helping to decide what research to do)
- ◯ Design of research (input into design and commenting on protocols, patient information sheets)
- ◯ Management of research (Advisory Boards / Steering Committees)
- ◯ Undertaking research (carrying out interviews, data analysis)
- ◯ Dissemination of findings (talking/writing about the results)
- ◯ Co-researcher
- ◯ Co-applicant
- ◯ Other (please specify)

10. How important is PPI in research to you?

- Very important
- Quite important
- Not especially important
- Not at all important
- No opinion
- It depends
- Other (please specify)

11. Why do you feel this level of importance about PPI in research?

12. How important is it to you that researchers give feedback (on your comments) to you personally?

- Very important
- Quite important
- Not especially important
- Not at all important
- No opinion
- It depends
- Other (please specify)

13. Why do you place this level of importance on receiving feedback?

14. Can you estimate how many research studies you have been involved with (giving PPI advice not as a study participant)? If it is a high figure, please just estimate.

15. In the last <u>five</u> research studies you have been involved with how many have you received feedback from the researchers about your comments?

16. Who have you received feedback from?

- A member of the research team
- The PPI co-ordinator / Lead (the person who runs the PPI group)
- Other
- for 'other ' or 'it depends' answers please explain

17. Do you generally receive feedback on your comments?

- Always
- Sometimes
- Never - if never please skip to Q.25 towards the end of the survey to leave your comments

Comments

18. Do you feel that the feedback you receive is timely?

- Yes
- Not applicable
- No (please state how the period of time you would have liked to receive comments in)

19. In general, when you make comments on research documents or provide other contributions, what sort of feedback do you receive either from the researchers or through your PPI Lead? You may tick more than one.

- [ ] I do not hear anything
- [ ] I do not hear from the researcher directly but through the PPI co-ordinator/lead (person who runs the PPI group)
- [ ] They acknowledge my comments have been received
- [ ] They let me know my comments were useful
- [ ] They let me know my comments led to changes/modifications (not detailed)
- [ ] They let me know my comments led to changes/modifications and these are detailed (i.e. track changes on an information leaflet, sent new copy of document)
- [ ] They let me know why they did not use my comments
- [ ] They let me know they would like more comments
- [ ] We have a dialogue/conversation (back and forth) about my comments
- [ ] Not applicable
- [ ] Other (please specify)

20. In general, when you make comments on research documents or provide other contributions, which sort of feedback is the <u>most common</u>?

- ○ I do not hear anything
- ○ I do not hear from the researcher directly but through the PPI co-ordinator/lead (person who runs the PPI group)
- ○ They acknowledge my comments have been received
- ○ They let me know my comments were useful
- ○ They let me know my comments led to changes/modifications (not detailed)
- ○ They let me know my comments led to changes/modifications and these are detailed (i.e. track changes on an information leaflet, sent new copy of document)
- ○ They let me know why they did not use my comments
- ○ They let me know they would like more comments
- ○ We have a dialogue/conversation (back and forth) about my comments
- ○ Not applicable
- ○ Other (please specify)

21. How do researchers let you know their feedback? You may tick more than one.

- [ ] Email
- [ ] Telephone
- [ ] Face to face
- [ ] Letter / paper
- [ ] Not applicable
- [ ] Other (please specify)

22. How would you like to receive feedback?

23. In your last project how satisfied were you with the feedback you received?

- ( ) Very satisfied
- ( ) Fairly satisfied
- ( ) Neither
- ( ) Fairly unsatisfied
- ( ) Very unsatisfied
- ( ) Not applicable

24. In general, how satisfied are you with the feedback you receive?

- ( ) Very satisfied
- ( ) Fairly satisfied
- ( ) Neither
- ( ) Fairly unsatisfied
- ( ) Very unsatisfied
- ( ) Not applicable

25. Why do you think researchers do not provide feedback?

26. What do you think is good feedback to PPI representatives?

27. How can PPI feedback be improved?

28. Have you noticed any changes in feedback on your contributions from researchers in the last 12 months?

- ◯ Yes it's changed, improved
- ◯ Yes it's changed, got worse
- ◯ No change
- ◯ It varies (please provide details below)
- ◯ I have not had any involvement in the last 12 months
- ◯ I don't know

29. Please provide further details explaining your response above, such as were any changes to the quality or quantity of feedback:

30. Please provide any other comments if you wish:

Please do not copy or reproduce this questionnaire without permission from the authors.
