# Supplementary material for: The role of patient and public involvement leads in facilitating feedback: “invisible work”
Source: Res Involv Engagem. 2020 Jul 10;6:40. doi: 10.1186/s40900-020-00209-2 (PMC7353750; doi:10.1186/s40900-020-00209-2)
Supplement: Supplementary file 2 — Additional file 2. Topic guide. [file 40900_2020_209_MOESM2_ESM.docx]

**PPI Feedback Study: Interview/Focus group questions for Extension (Stage 2)**

**1. PPI Coordinator/Leads**

You have been part of the ‘Completing the feedback study’ for the last year and a half:

**Can you tell me a little about how feedback was communicated to your PPI representatives before the creation and distribution of the group-specific feedback tool?**

We then created a local group tool during a meeting in November 2016.

**Can you recall how the tool was finalised after that meeting and distributed to the researchers using that group?**

**Has feedback changed since that feedback tool was designed and implemented?**

**If so – how?**

**If not – why do you think that is?**

**What processes did you use to encourage researchers to use the feedback tool?**

**Were the researchers reminded to use the feedback tool?**

**If so, when?**

**If not, why not?**

**How was the researcher feedback then communicated to the PPI representatives?**

**Do you get a sense of how that tool was received by researchers?**

**Why might that be?**

**How might things have been done differently?**

**Have you noticed any problems that the researchers have had in using the form?**

**Do you get a sense of how that tool has changed feedback received by PPI representatives?**

**Why might that be?**

**How might things have been done differently?**

**Do you get a sense of how PPI representatives feel about researchers using/not using the tool?**

**Has using the tool been of wider benefit to your group? To you as the Lead or to the researchers or PPI representatives?**

**Has using the tool had any detrimental effects to the group? To you as the Lead or to the researchers or PPI representatives?**

**Were there any organisational/institutional challenges that you faced in implementing the PPI feedback tool?**

In July we created one regional feedback tool/Guidance. **How did you find the process of [trying to] design a single tool?**

**Which tool have you been using in your group?**

**Why is that?**

**Have the researchers responded differently to the two forms?**

**2. Researchers**

**What are your opinions on providing feedback to PPI representatives you work with?**

**(How) do you feedback your comments to the PPI representatives you work with?**

**Are you aware they your group does/doesn’t use a feedback tool to help you to feedback your comments to your PPI representatives?**

[If they use the tool]

**What do you think about this feedback tool/process?**

**Has using the tool/process changed the way that you think about feedback?**

**If so, why do you think that is?**

**Has using this tool been of wider benefit to you?** PROMPT i.e. the way that you think about PPI representatives or about your research or about the research process in general.

[If they don’t use the tool]

**What barriers have there been to you using the feedback tool/s?**

**What could be done to improve this tool/process?**

[If they have used both the group-specific and regional tool]

**Which tool did you find the easiest to use?**

**Why was this?**

**What could be done to improve the tool/s?**

[if they don’t have a tool I their group]

**Are you aware that some PPI groups in the EoE have a feedback tool/Guidance to help you to feedback your comments to your PPI representatives?**

**How do you feel about using a tool like this**?

**3. PPI Representatives**

**How long have you been working as a PPI representatives with X group/s?**

**How do you receive feedback on the work you have done for a researcher?**

[if their group has a tool]

**Since the implementation of feedback tool/s, have you noticed a difference to the [type, quality and frequency of the] feedback you receive?**

**Has this changed the way that you feel about working with researchers**?

**Has this changed the way that you feel about working as a PPI representative?**

**How do you think that the tool has been received by researchers you work with?**

**How do you think the tool has been received by other PPI representatives in the group?**

**What could be done to improve the tool/process?**

**Has there been anything negative about the use of the tool/process?**

**Are you aware of any organisational/institutional challenges that existed in implementing the PPI feedback tool?**

[if their group has NOT got a tool]

**Are you aware that some PPI groups in the EoE have a feedback tool/Guidance to help researchers feedback their comments to their PPI representatives?**

**How do you feel about using a tool like this**?
