# Supplementary material for: The role of patient and public involvement leads in facilitating feedback: “invisible work”
Source: Res Involv Engagem. 2020 Jul 10;6:40. doi: 10.1186/s40900-020-00209-2 (PMC7353750; doi:10.1186/s40900-020-00209-2)
Supplement: Supplementary file 3 — Additional file 3. GRIPP2 checklist. [file 40900_2020_209_MOESM3_ESM.docx]

Additional File 3: GRIPP2 checklist

| Section and topic | Item | Reported on page No |
| --- | --- | --- |
| 1: Aim | Report the aim of PPI in the study  *PPI is central to this paper and the aim was to address a concern raised by PPI contributors and influence/improve feedback. The PPI contributors have been involved throughout. Full details of PPI within this study have already been reported in earlier papers, so the full details are not repeated but referred to throughout the paper (reference 8 and 23).*  Mathie, E., et al. *Reciprocal relationships and the importance of feedback in patient and public involvement: A mixed methods study*. Health Expectations, 2018. **21(5)**, 889-908.  Mathie, E., et al., *Regional working in the East of England: using the UK National Standards for Public Involvement.* Research Involvement and Engagement, 2018. **4:48**. | p.3 |
| 2: Methods | Provide a clear description of the method used for PPI in the study  *PPI contributors were involved throughout: they initiated the idea for the study, designed, carried out data collection, data analysis and have been involved in disseminating throughout. Two of the PPI contributors (DM, GR) have been involved in writing this paper, making comments, making revisions and writing the lay summary.* | p.3 |
| 3: Study results | Outcomes—Report the results of PPI in the study, including both positive and negative outcomes  *PPI contributors have shaped the research and were part of the process of changing the feedback processes, these are described in the paper. Co-design of feedback forms and processes are outlined in section Co-designing Feedback Processes.*  *The positive and negative outcomes were detailed in the earlier paper and is referenced in this paper (reference 23).* | p.6 |
| 4: Discussion and conclusions | Outcomes—Comment on the extent to which PPI influenced the study overall. Describe positive and effects  *PPI influenced the overall study.* | p.12 |
| 5: Reflections/critical perspective | Comment critically on the study, reflecting on those that went well and those that did not, so others can learn from this experience  *This paper is linked to Mathie et al (2018) [reference 23] where reflections (positive and negative) on PPI are reported.* | p.12 |

PPI=patient and public involvement
