# Supplementary material for: The role of patient and public involvement leads in facilitating feedback: “invisible work”
Source: Res Involv Engagem. 2020 Jul 10;6:40. doi: 10.1186/s40900-020-00209-2 (PMC7353750; doi:10.1186/s40900-020-00209-2)
Supplement: Supplementary file 4 — Additional file 4. Researcher Feedback Form 2018. [file 40900_2020_209_MOESM4_ESM.doc]

| Organisation LOGO | **Public Involvement Group**  **Researcher Feedback Form** |
| --- | --- |

Dear Researcher(s)

To allow us to evaluate the contribution and impact of the [PPI group] on research, please complete the following feedback form. We value your feedback and really appreciate knowing how helpful our comments were (or not) and whether any changes to the research proposal or documents you asked us to review were made as a result of these comments. Your completion of this form will also be helpful to you when completing the Patient and Public Involvement (PPI) sections of funding application forms and providing project updates if funded.

| Project Title: |  |
| --- | --- |
| Name and contact details of lead researcher (including institution & email address): |  |
| 1. Overall, how helpful were the comments received from [PPI group] members? | **Please circle/underline one response:**  Very helpful Quite helpful Not helpful |
| 1. Has the review from [PPI Group] informed and/or influenced the development of your research application/ideas? | **Please circle/underline one response:**  Yes No |
| 3a) **If you answered YES to Q2**, please detail how comments from the [PPI group] informed and /or influenced your research. | **Please give details of specific changes made to your study/research documents:**  **PTO** |
| 3b) **If you answered NO to Q2**, please detail why you think the comments received from the [PPI group] haven’t informed and /or influenced your research. | **Please outline the reasons why you didn’t make any changes to your study/research documents:** |
| 4) Would you recommend the [PPI group] to colleagues? | **Please circle/underline one response:**  Yes No Not sure  . |
| 5) Do you have any comments or suggestions regarding how to improve the process of consulting/collaborating with the [PPI group]? | **Please enter any suggestions or comments here:** |

**Please complete the following:**

**Form completed by:**

**Date you plan to submit proposal/ethics application (please specify):**

**Date you expect to hear whether successful/not successful:**

**Thank you for completing this feedback form.**

If you have completed this form electronically or can scan the form, please return to [xxxxx]. If you completed a hard copy version, please return this form by post to: xxxxxx

**Funding statement:**

This was developed as part of independent research funded by the National Institute for Health Research (NIHR) Collaborations for Leadership in Applied Health Research and Care East of England (CLAHRC EoE now ARC EoE) Programme.

**NIHR disclaimer statement:**

The views expressed are those of the author(s) and not necessarily those of the NHS, the NIHR or the Department of Health and Social Care.

***PLEASE DO NOT COPY OR REPRODUCE THIS DOCUMENT WITHOUT PERMISSION FROM THE RESEARCHERS E.J.Mathie@herts.ac.uk***
